# Development of an advanced liquid chromatography–tandem mass spectrometry measurement system for simultaneous sphingolipid analysis

**DOI:** 10.1038/s41598-024-56321-w

**Published:** 2024-03-08

**Authors:** Baasanjav Uranbileg, Eri Sakai, Masayuki Kubota, Hideaki Isago, Masahiko Sumitani, Yutaka Yatomi, Makoto Kurano

**Affiliations:** 1 Department of Clinical Laboratory Medicine, Graduate School of Medicine, The University of Tokyo, 7-3-1 Hongo, Bunkyo-ku, Tokyo, 113-8655 Japan; 2 Nihon Waters K.K., Tokyo, Japan; 3 Department of Pain and Palliative Medicine, The University of Tokyo Hospital, Tokyo, Japan

**Keywords:** Sphingolipid, Ceramide1-phosphate (Cer1P), Hexosylceramide (HexCer), Lactosylceramide (LacCer), dh-ceramide, Deoxy-ceramide, Deoxy-dh-ceramide, Biochemistry, Metabolomics

## Abstract

Mass spectrometry-based lipidomics approaches offer valuable tools for the detection and quantification of various lipid species, including sphingolipids. The present study aimed to develop a new method to simultaneously detect various sphingolipid species that applies to diverse biological samples. We developed and validated a measurement system by employing a single-column liquid chromatography-mass spectrometry system utilizing a normal-phase separation mode with positive ionization. The measurement system provided precision with a coefficient of variant below 20% for sphingolipids in all types of samples, and we observed good linearity in diluted serum samples. This system can measure the following sphingolipids: sphingosine 1-phosphate (S1P), sphingosine (Sph), dihydroS1P (dhS1P), dihydroSph (dhSph), ceramide 1-phosphate (Cer1P), hexosylceramide (HexCer), lactosylceramide (LacCer), dh-ceramide, deoxy-ceramide, deoxy-dh-ceramide, and sphingomyelin (SM). By measuring these sphingolipids in cell lysates where S1P lyase expression level was modulated, we could observe significant and dynamic modulations of sphingolipids in a comprehensive manner. Our newly established and validated measurement system can simultaneously measure many kinds of sphingolipids in biological samples. It holds great promise as a valuable tool for laboratory testing applications to detect overall modulations of sphingolipids, which have been proposed to be involved in pathogenesis processes in a series of elegant basic research studies.

## Introduction

Liquid chromatography–tandem mass spectrometry (LC–MS/MS) based lipidomics approaches offer valuable tools for the detection and quantification of various lipid species^1^. LC–MS/MS has become a widely used technique in various fields, including medical science, due to its high sensitivity, selectivity, and capability for structural elucidation, which make it a powerful tool for the analysis of complex samples such as human serum^2–5^, cerebrospinal fluid (CSF)^6–8^, urine^9–11^, ascites^12^, and aqueous humor^13^.

A vast amount of basic studies have described the importance of biologically active lipids as mediators in various human diseases such as cancer^14,15^, immunological disorders^16^, cardiovascular disorders^17,18^ and neurological disorders^19^. Among biologically active lipid mediators, sphingolipids have received significant attention not only as components of biological structures but also due to their involvement in the aforementioned human diseases. Sphingolipids, such as sphingosine 1-phosphate (S1P), sphingosine (Sph), and ceramides (Cer), are emerging as potential treatment targets based on their involvement in disease pathophysiology, as proposed by a series of elegant basic studies^16,20–23^. To further understand and validate their involvement in human disease, it is essential to determine the levels of sphingolipids in human samples. By accurately quantifying and characterizing these sphingolipids, we can facilitate translational research to develop novel laboratory testing and/or identify novel therapeutic targets.

The first report of LC–MS/MS for the quantitative analysis of sphingolipids using electrospray ionization was made in 1997^24^, but there was a residual issue with the broadness of the S1P peak. Later, a more sensitive, reliable, and upgraded method was introduced^25^, although it could measure only S1P and Sph. Another group developed a method for the quantification of sphingolipids with a more sensitive ultra-performance LC–MS/MS method^26^, although this method requires very complicated sample preparation. Recently, we developed a rapid, highly specific, and sensitive method that requires only a simple sample preparation and simultaneously measures six important sphingolipids in biological samples^6,9^.

**Figure 1:**
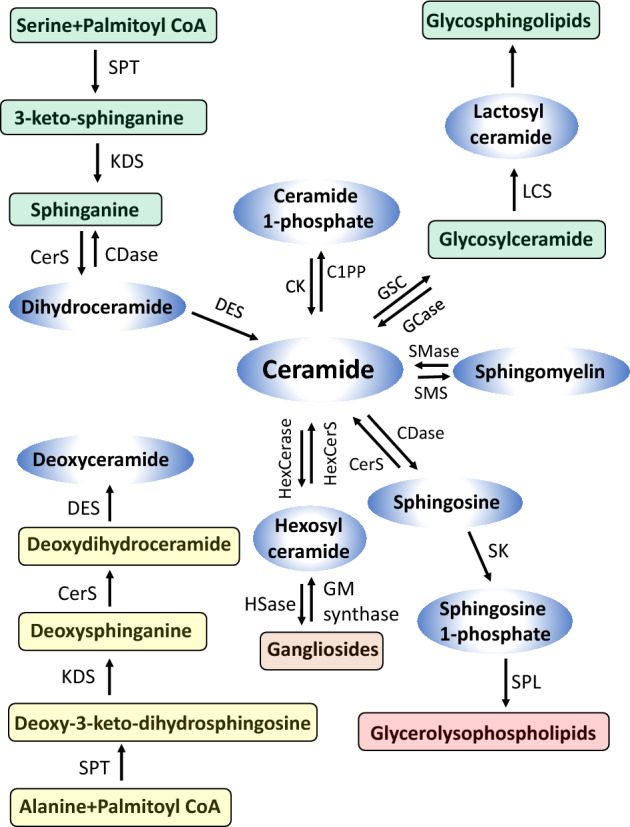
Pathways of sphingolipid metabolism. Sphingolipids have three major metabolic pathways: the de novo pathway, which originates from saturated fatty acids, the salvage pathway, and the sphingomyelin pathway. All of these pathways converge in ceramides. *CPT* serine palmitoyltransferase, *KDS* 3 ketosphinganine reductase, *CerS* ceramide synthase, *CDase* ceramidase, *DES* dihydroceramide desaturase, *CK* ceramide kinase, *C1PP* ceramide 1-phosphate phosphatase, *GSC* glucosylceramide synthase, *GCase* glycosidase, *LCS* lactosyl ceramide synthase, *SMase* sphingomyelinase, *SMS* sphingomyelin synthase, *SK* sphingosine kinase, *SPL* S1P lyase, *HexCerase* hexosylceramidase, *HexCerS* hexosylceramide synthase, *HSase* hexosaminidase.

In the previous methods mentioned, one of the main limitations was the restricted number of sphingolipids that could be measured simultaneously. As shown in Fig. 1, lipids belonging to sphingolipids can be converted into each other, forming a dynamic and complicated metabolic map. Therefore, comprehensive analyses of these sphingolipids are desirable to better understand the modulations and involvement of sphingolipids in human disease. In this study, our primary objective was to develop a novel method for the analysis of more species of sphingolipids, such as ceramide 1-phosphate, hexosylceramide, lactosylceramide, dh-ceramide, deoxy-ceramide, and deoxy-dh-ceramide. We aimed to establish a method to analyze these comprehensive sphingolipids in a wide range of biological samples, including serum, CSF, urine, and cell and tissue lysates.

## Results

### Establishment and validation of the sphingolipid measurement system based on LC–MS/MS

**Figure 2:**
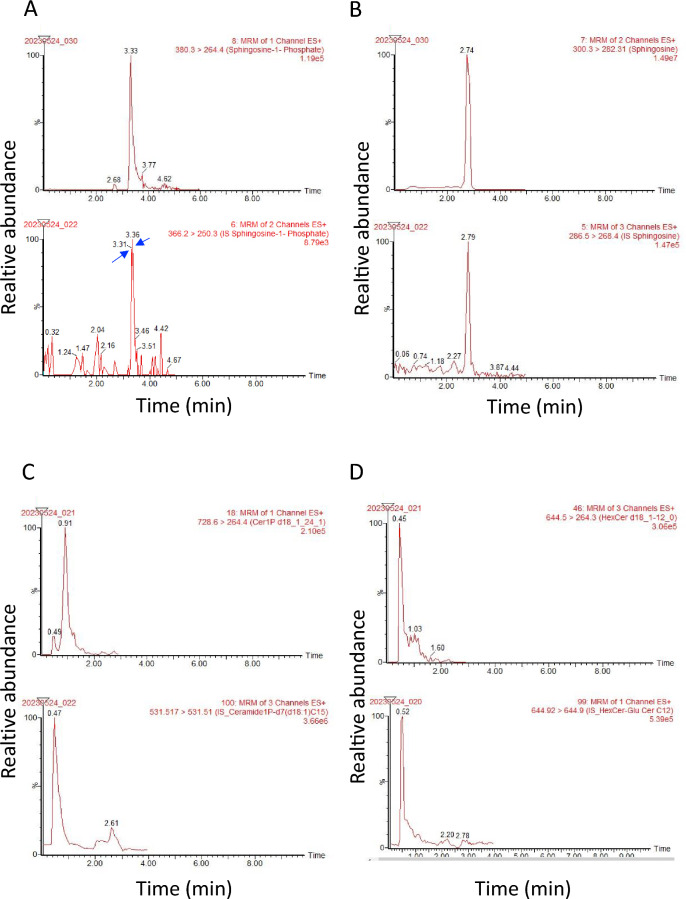
Retention times and chromatograms obtained from the MRM transitions. Representative samples' retention times (X axis) and extracted ion chromatograms (Y axis, relative abundance) of key sphingolipids, including sphingosine 1-phosphate (**A**), sphingosine (**B**), ceramide 1-phosphate 24:1 (**C**), and hexosylceramide 12:0 (**D**). The upper panels show chromatograms of the representative samples, while the lower panels display chromatograms of internal standards. The retention times of the peaks corresponding to each compound are indicated on the X axis. The Y axis represents the relative abundance of each compound as detected by mass spectrometry. These chromatograms provide visual representations of the separation and detection of sphingolipid species using the MRM transitions.

**Figure 3:**
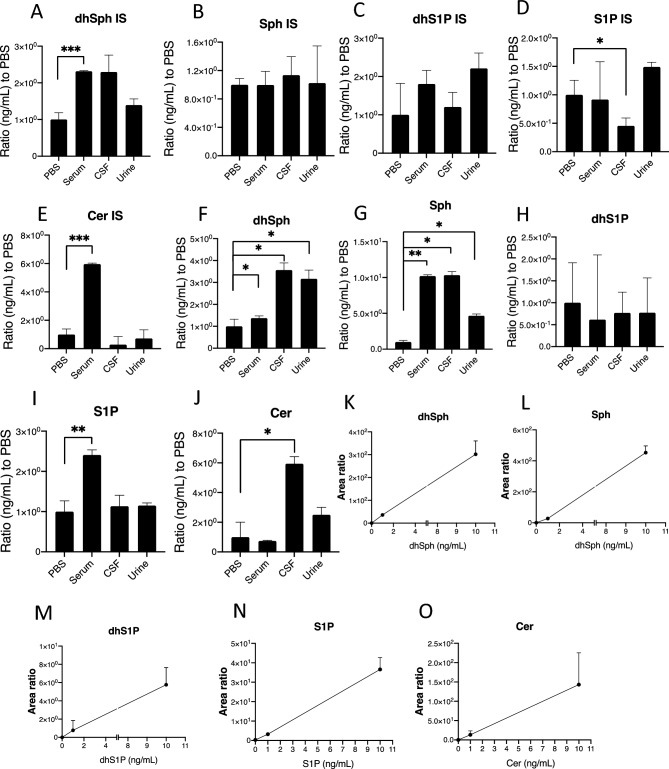
Matrix effect in serum, cerebrospinal fluid, and urine samples. **(A–E)** To thoroughly investigate potential matrix effects, 10 μL of internal standards were prepared at a precisely controlled concentration of 1.0 ng/mL and combined with 10 μL of various matrices, including PBS, serum, cerebrospinal fluid (CSF), and urine samples. Peak areas of internal standards (C17S1P and C17dhS1P) were compared with additional internal standards (C17Sph, C17dhSph, and Cerd18:1/17:0) across n = 5 replicates for robust statistical analysis. Results are presented as ratios relative to the average peak of internal standards added to PBS. Statistical significance is indicated as *P < 0.05, **P < 0.01, and ***P < 0.001. (**F–J)** To assess potential matrix effects, the peak area of sphingosine 1-phosphate (S1P), dihydro-S1P (dhS1P), sphingosines, or ceramides in samples was subtracted from samples spiked with standard S1P, dhS1P, sphingosines, or ceramides at a concentration of 1 ng/mL. Subsequently, the peak areas of S1P, dhS1P, sphingosines, or ceramides were compared across n = 5 replicates to ensure statistical robustness. The resulting data are presented as ratios relative to the average peak area of internal standards added to PBS, facilitating clear interpretation. Significance levels are indicated as *P < 0.05 and **P < 0.01. (**K–O)** Linearity analysis of sphingolipids. To validate the measurement of dihydro-sphingosine (dhSph), sphingosine (Sph), dihydro-S1P (dhS1P), S1P, and ceramides, standard mixtures were prepared at three concentrations (0.0 ng/mL, 1.0 ng/mL, and 10 ng/mL) in PBS. Measurements were conducted and assessed for linearity using a range of standards, including Ceramide (d18:1/18:0), S1P (d18:1), dhS1P (18:0), Sph (d18:1), and dhSph (d18:0). This analysis offers valuable insights into the reliability and accuracy of measurements across different concentrations of sphingolipids, ensuring robustness in the measurement methodology.

**Figure 4:**
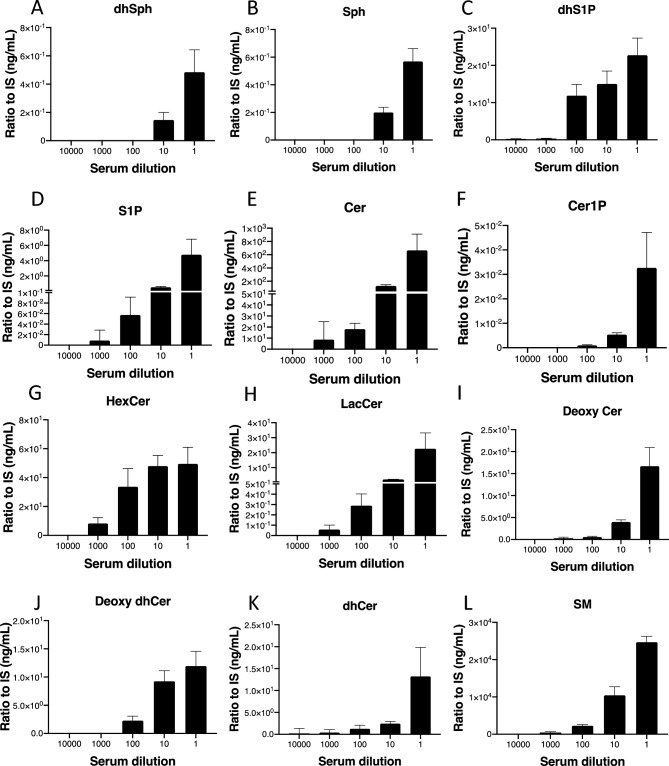
Linear regression analysis of sphingolipids in serially diluted serum samples. A comprehensive investigation was conducted using linear regression analysis encompassing all 12 sphingolipids: dhSph (**A**), Sph (**B**), dhS1P (**C**), S1P (**D**), Cer (**E**), Cer1P (**F**), HexCer (**G**), LacCer (**H**), deoxy cer (**I**), deoxy dhCer (**J**), dhCer (**K**), and SM (**L**) in serially diluted serum samples. The analysis, executed following a well-established method, involved five distinct steps. To ensure robustness and reliability, each step was meticulously repeated five times, providing a thorough and reproducible examination of the sphingolipid composition in the serum samples.

First, we developed the sphingolipid measurement system based on LC–MS/MS, with which we could detect ceramides (Cer), sphingomyelins (SM), ceramide 1-phosphate (Cer1P), hexosyl ceramide (HexCer), lactosylceramide (LacCer), dihydroceramide (dhCer), deoxy-ceramide (Deoxy Cer), deoxy-dihydro-ceramide (deoxy dhCer), sphingosine 1-phosphate (S1P), dihydroS1P (dhS1P), sphingosine (Sph), and dihydrosphingosine (dhSph). Extracted ion chromatograms obtained from the MRM transitions are shown in Fig. 2 and Supplementary Fig. S1. We could clearly determine the peaks of S1P, dhS1P, Cer1P, HexCer, dhSph, Cer, SM, and dhS1P in serum, CSF, and urine samples (Suppl. Fig. 2S). The interday and intraday precisions determined with PBS containing 1.0 ng/mL and 10 ng/mL standard mixtures and the plasma, CSF, and urine samples are shown in Table 1. The CVs for all types of samples were nearly or below 20%, and most of the CVs were above 15% for the sphingolipids which were detected by this method for the first time as HexCer, SM, Deoxy dhCer. Almost all CVs were below 20%, suggesting that sufficient reproducibility was achieved to measure all these sphingolipid levels in the serum, CSF, and urine using this system. We observed no obvious matrix effects for internal standards in the urine samples (Fig. 3A–E). The signals for C_17_Sph and d18:1/17:0 Ceramide were augmented in the serum samples (Fig. 3A, E), and those of C_17_S1P were augmented in the CSF samples (Fig. 3D). For endogenous sphingolipids, we observed that the signals for dhSph and Sph were significantly increased in all three types of samples, while those of S1P were augmented only in serum samples, and those of Cer were augmented in CSF samples (Fig. 3F–J). However, we confirmed linearity over the range of tested concentrations of standard solutions (0.0 ng/mL, 1.0 ng/mL, 10.0 ng/mL) in PBS, as depicted in Fig. 3K–O. Linearity analysis using standard solutions in serum, CSF, and urine samples was also conducted, as shown in Supplementary Figure S3 (A-C). Moreover, we also measured all sphingolipids in diluted serum samples based on a high concentration of the lipids. Figure 4A–L describes good linearity in levels of the sphingolipids measured in the series of diluted serum samples.

**Table 1:** Precision of the method for sphingolipids.

Sphingolipids	dhSph	dhS1P	Sph	S1P	Total Cer1P	Total Cer	Total HexCer	Total SM	Total LacCer	Total dhCer	Total deoxy dhCer	Total deoxy Cer
Inter-day												
Serum	18.4	10.1	11.7	16.1	10.3	15.6	16.0	19.2	18.2	9.6	15.5	17.6
CSF	10.4	4.5	10.0	4.4	10.0	9.2	12.9	14.8	14.5	7.9	20.2	9.7
Urine	18.9	5.2	14.8	7.7	13.8	9.6	19.9	16.6	15.0	14.3	16.2	15.3
1 ng/mL in PBS	18.3	8.2	15.7	17.5	15.3	14.9	19.5	16.1	–	16.1	7.9	20.0
10 ng/mL in PBS	17.5	18.9	12.2	14.6	5.1	18.3	19.3	16.5	–	8.4	16.4	15.3
Intra-day												
Serum	8.7	14.5	15.4	9.6	12.7	18.0	16.9	10.2	7.2	16.9	19.8	4.3
CSF	13.9	16.5	15.7	14.4	19.1	20.7	19.7	19.9	10.2	18.2	16.2	15.5
Urine	15.8	19.3	9.6	17.4	11.0	19.4	19.9	14.3	6.2	14.9	16.9	5.7
1 ng/mL in PBS	13.7	–	15.2	15.3	16.8	12.1	12.4	–	–	20.1	19.5	–
10 ng/mL in PBS	17.0	–	18.2	16.7	13.1	10.0	–	12.3	–	19.3	17.4	3.7

### The levels of sphingolipids in diverse types of biological samples

**Figure 5:**
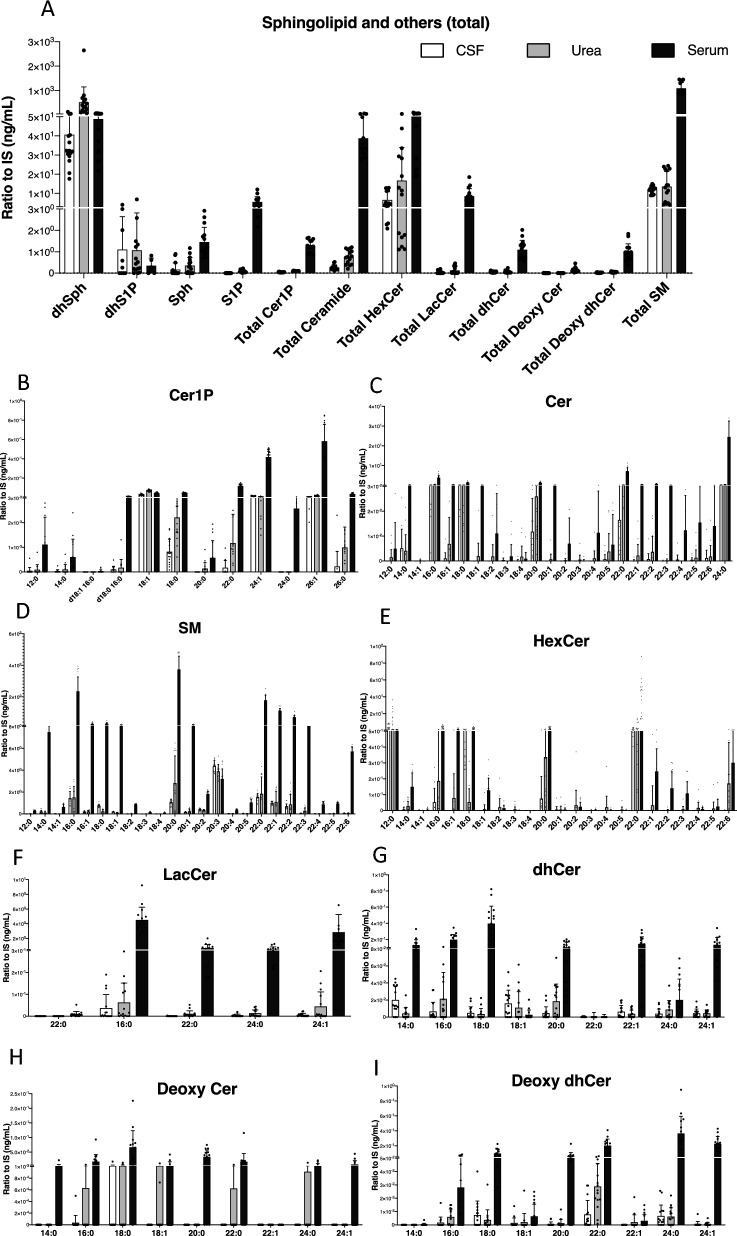
Sphingolipid levels in human serum, CSF, and urine samples. **(A)** This analysis involved quantifying the levels of dhSph, dhS1P, Sph, and S1P, as well as the total levels of Cer1P, Cer, HexCer, LacCer, dhCer, deoxy cer, deoxy dhCer, and SM in human cerebrospinal fluid (CSF, white column), urine (pale grey column), and serum (grey column) samples. Each sample type comprised n = 5 measurements, and each measurement was repeated three times to ensure accuracy and reliability. The presented results represent the mean ± standard deviation (SD) of the measurements, providing a comprehensive overview of sphingolipid levels across different sample types. (**B–I**) For each species of Cer1P, Cer, HexCer, LacCer, dhCer, deoxy cer, deoxy dhCer, and SM, levels were determined in human CSF (white column), urine (pale grey column), and serum (grey column) samples. The analysis encompassed n = 5 samples from each type, with each sample measured three times for accuracy and reliability. The reported results are presented as the mean ± SD, offering a comprehensive overview of the variations in individual sphingolipid species across different biological samples.

As a newly developed and validated method for the simultaneous measurement of a wide range of sphingolipids, next, we examined sphingolipid levels in serum, CSF, and urine samples. Figure 5 shows the results of sphingolipids in serum, CSF, and urine, including the total levels of sphingolipids (Fig. 5A) and the levels of each species (Fig. 5B–I). The levels of dhSph, total HexCer, and total SM were abundant in all types of samples but were dominant in the serum sample, especially in the case of the latter two. dhS1P levels were higher in CSF and urine samples, but when considering all measurable sphingolipids by this method, they were found to be extremely rich in the serum sample (Fig. 5A).

We could detect expanded species for Cer (total of 24 species) and SM (total of 23 species) where most of the species were at higher levels in serum than those in CSF and urine samples (Fig. 5C, D). Additionally, measured Cer1P (total of 12 species), HexCer (total of 23 species), LacCer (total of 5 species), dhCer, Deoxy Cer, and Deoxy dhCer (9 species each) were also most abundant in serum samples (Fig. 5B, E–I). These results suggested that this method might enable us to determine the actual levels of these lipids in various biological samples. Additionally, it provided insights into the baseline levels of these lipids in these sample types.

### Sphingolipid levels were found to be altered as a result of the modulation of S1P lyase (SPL)

**Figure 6:**
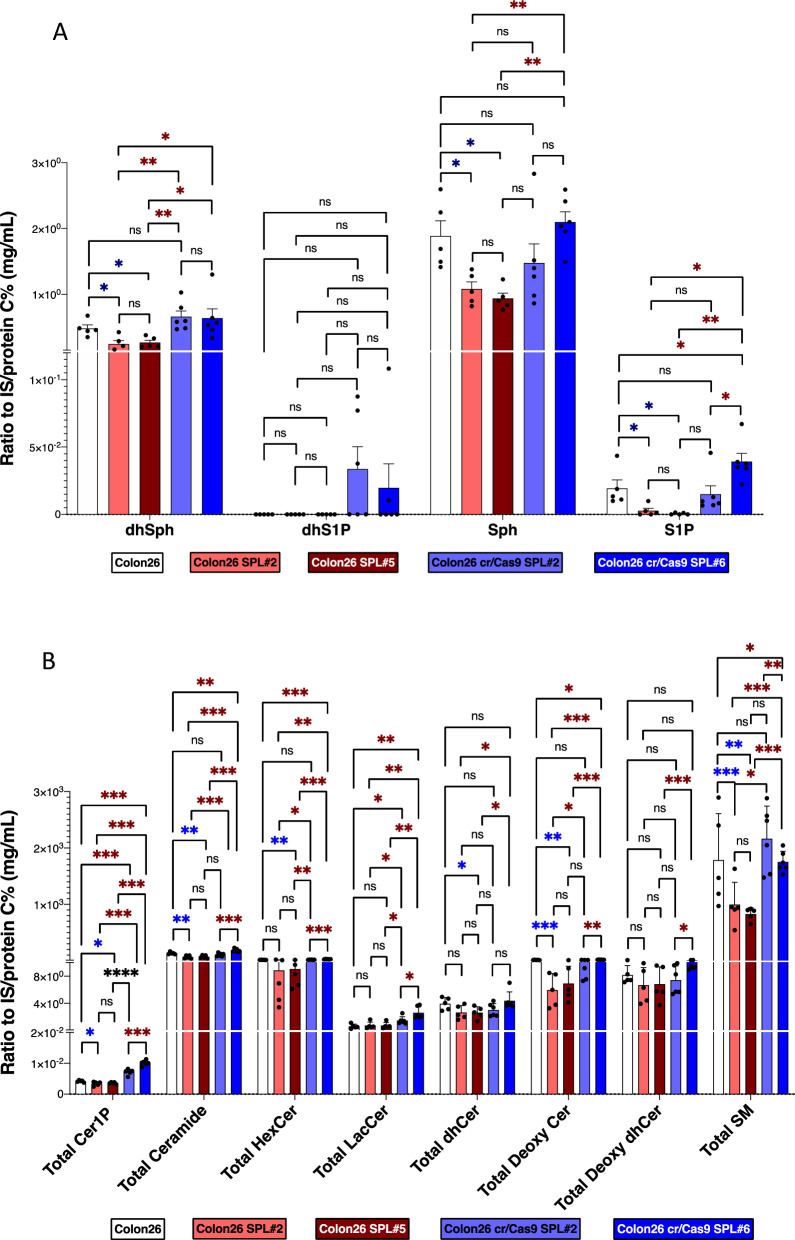
Alterations in sphingolipid levels due to SPL modulation. **(A)** This detailed analysis explores the levels of dihydro-sphingosine (dhSph), dihydro-S1P (dhS1P), sphingosine (Sph), and S1P across different cell lines. The investigated cell lines include control Colon26 cells (white column), SPL-overexpressing cells (pink column—moderately expressed #2, cherry red column—highly expressed #5), and SPL-inhibited cells (light blue #2 and blue columns #6). Each condition was evaluated using n = 5 samples, with measurements meticulously repeated to ensure reliability. The reported results are expressed as the mean ± standard deviation (SD), providing valuable insights into the dynamic variations in sphingolipid levels across diverse cellular states. (**B**) The total levels of Cer1P, Cer, HexCer, LacCer, dhCer, deoxy cer, deoxy dhCer, and SM were quantified in control Colon26 cells (white column), SPL-overexpressing cells (pink column—moderately expressed #2, cherry red column—highly expressed #5), and SPL-inhibited cells (light blue #2 and blue columns #6). Each condition comprised n = 5 samples, and the mean ± standard deviation (SD) was used to present the results. The p-values, denoted by *, **, or ***, indicate statistical significance (*p < 0.05, **p < 0.01, ***p < 0.001), with blue * representing a significant decrease and red * representing a significant increase in comparison to the control cell lines.

In the final experiment, to investigate whether this method can comprehensively elucidate the dynamic modulations of sphingolipids, we employed this method to measure sphingolipid levels in mouse colon cancer cell lysates with enhanced expression of S1P lyase (SPL) or its inhibition using the CRISPR/Cas9 system, as previously described^27^. We used 5 cell lines: Colon 26 as a control, Colon26 + SPL#2 (moderate) and Colon26 + SPL#5 (strong) as SPL-overexpressing cells, and Colon26 + Cr/SPL#2 and Colon26 + Cr/SPL #6 as SPL-inhibited cell lines. SPL is responsible for S1P degradation and irreversibly degrades it, shifting from a sphingolipid to a glycerolysophospholipid pathway^27^. Therefore, in addition to changes in S1P levels, it was worth measuring other sphingolipids and assessing the modulation of the sphingolipid pathway through SPL. Similar to our previous results, S1P levels decreased in SPL-overexpressed cells and increased in SPL-inhibited cell lines compared to the control cell line **(**Fig. 6A). There were no changes in dhS1P levels. Sph and dhSph exhibited similar trends to S1P in the cell lines. Figure 6B depicts the total levels of Cer1P, Cer, HexCer, LacCer, dhCer, Deoxy Cer, Deoxy dhCer, and SM, showing that SPL overexpression led to decreased levels of all these sphingolipids, while SPL inhibition resulted in their accumulation. Detailed levels of each species of these sphingolipids are shown in Supplementary Fig. S4A–F. These results support the role of SPL in sphingolipid pathway modulation in mouse colon cancer cell lysates with enhanced or inhibited SPL expression, demonstrating changes in S1P levels and corresponding alterations in other sphingolipids (Supplementary Fig. S4).

## Discussion

In this study, we developed and validated a measurement system by employing a single-column LC–MS/MS system utilizing a normal-phase separation mode with positive ionization in human serum, CSF, urine, and cell lysates. Previous studies have shown sufficient sensitivity for measuring sphingolipids^25,28,29^, but until recently^6,9^, we were unable to perform simultaneous measurements of S1P, dhS1P, Sph, and ceramides without changing the mobile solvents. To address these limitations, including difficulties in sample preparation and a restricted number of sphingolipids, we developed and validated a method using the same mobile solvent, column, and measurement conditions, but expanded the analysis to include a greater number of sphingolipid species. Sphingolipids encompass a wide range of molecules with crucial roles in biological structures that serve as key regulators of various cellular functions^21,23^. Given the significant impact of their structural attributes on biological activity and the dynamic metabolic map formed by various sphingolipids (Fig. 1), there is a pressing demand for comprehensive techniques to identify and quantify as many distinct species as possible. Considering already developed measurement systems for S1P, dhS1P, Sph, and dhSPh, this study evaluated a method to simultaneously detect sphingolipids as follows: Cer1P (12 species), Cer (24 species), SM (23 species), HexCer (23 species), LacCer (5 species), dhCer, Deoxy Cer and Deoxy dhCer (9 species each) (Fig. 5).

These sphingolipids have significant implications in both normal physiological processes and pathological conditions. Cer1P, in conjunction with S1P, is closely linked to cell fate. The delicate balance between them, known as the “sphingolipid rheostat,” holds crucial importance in diverse functions within eukaryotic cells^30,31^. dhCer, considered a complex sphingolipid, undergoes modification to ceramide, playing a notable role in cellular processes^32^. Deoxy Cer is recognized for its involvement in hereditary sensory autonomic neuropathy and type 2 diabetes mellitus, highlighting its relevance in specific medical conditions^33,34^. HexCer is a group of ceramide metabolites that have a neutral sugar moiety linked to ceramide and are connected to multidrug resistance in multiple cancers^35^. LacCer is a member of a large family of small molecular weight compounds known as glycosphingolipids and contributes to inflammation, atherosclerosis, skin conditions, hair graying, cardiovascular disease, and diabetes due to mitochondrial dysfunction^36^. With our new method, it is now possible to simultaneously measure these sphingolipids, allowing for the determination of their additional roles in both normal and pathological states across various samples.

Moreover, this method can help us understand the dynamic modulations of sphingolipid metabolism in pathological conditions. Alterations in all measurable sphingolipids were observed when we measured SPL overexpressing or SPL-inhibited cell lysates in comparison to control cells (Fig. 6 and Suppl. Fig. S5). As predicted and reported previously^27^, S1P levels increased in SPL-inhibited cells, while its levels decreased in SPL-overexpressing cell lines. Since many sphingolipids possess significant biological properties, as described above, measuring a single or a few sphingolipids might be insufficient to understand the significance of sphingolipids in human diseases. Therefore, we believe that this new method can be a powerful tool to understand overall modulations in sphingolipids and reveal their significance in the pathogenesis of human diseases.

The main limitation of the present method concerns matrix effects. As shown in Fig. 3, the signals of internal standards and some endogenous sphingolipids were differently influenced by the CSF and serum matrixes. Since the signal of S1P was suppressed in the matrix of CSF samples, its levels were underestimated (Fig. 5A). In serum samples, the levels of ceramides were overestimated. Regarding these matrix issues, we performed additional measurements and achieved good linearity when we used different concentrations of standards or serially diluted serum samples (Fig. 3K–O, Suppl. Fig. S3, Fig. 4). To measure the absolute concentration of the sphingolipids in the future, a stable isotope should be used as an internal standard, to align the matrix effects of the target lipids and internal standard, as we did to measure lysophosphatidylcholine levels^37^. Another minor limitation is CV values in inter-day and intra-day measurements (Table 1), especially HexCer, dhCer, Deoxy Cer. Deoxy dhCer showed CV values near 20%. Although we believe that we could understand the overall dynamic modulations of sphingolipids with this method, some additional improvements must be implemented to increase the precision of the method and approach CVs below 15%. Another limitation of this research pertains to the modulation of SPL expression levels in mouse colon cancer cell lines. Building upon insights from our previous investigation^27^, which delved into the role of SPL in cancer pathophysiology and acknowledged the interconverting properties of sphingolipids, we quantified and proposed comprehensive modulations of sphingolipids. These modulations are positioned upstream of S1P, which serves as the principal target of SPL, contingent upon its expression levels. To establish the specific and direct effects, further studies, such as confirmation of each enzyme activity within the sphingolipid pathway, are warranted.

In the present study, we measured sphingolipids in various human samples, including serum, CSF, and urine (Fig. 5). In general, most sphingolipid levels were abundant in human serum samples in comparison to CSF and urine samples, except dhSph, the level of which was extremely high in urine samples. Although the underlying mechanism for these differences remained unknown, by measuring sphingolipids in serum, CSF, and urine, we believe that we can understand their modulations and potential significance in the pathogenesis of human diseases more comprehensively, as we could in SPL-modulated cells (Fig. 6).

In conclusion, our newly developed measurement system can simultaneously measure multiple sphingolipids in biological samples. It holds great promise for laboratory testing, allowing the overall detection of comprehensive and dynamic changes in sphingolipid levels. This method will provide a powerful tool for further studies to evaluate sphingolipid abnormalities in various health conditions and across a variety of biological samples.

## Materials and methods

### Samples

Serum and urine samples were collected from healthy Japanese adult volunteers [female/male = 1/2, and age (mean ± S.D.) = 36.3 ± 3.3] who were not receiving any medication, after obtaining their informed consent. CSF samples were obtained from residual specimens from other laboratory examinations collected by lumbar puncture from Japanese subjects who were not diagnosed as having any disorders [female/male = 1/2, and age (mean ± S.D.) = 70.3 ± 9.1]. The collected samples were stored at − 80 °C until the measurements and were handled on ice during the experiments. This study was conducted with the approval of the Institutional Research Ethics Committee of the Faculty of Medicine of the University of Tokyo (11158 and 10516).

The mouse colon cancer cell line, Colon 26, was obtained directly from the Cell Resource Center for Biomedical Research, Tohoku University (TKG 0518). Cells were maintained in RPMI-1640 (189-02025, Fujifilm Wako Junyaku Co., Ltd., Osaka, Japan) containing 2000 mg/L glucose, 10% fetal bovine serum (FB-1280/500, Biosera, Fujifilm Wako Junyaku Co., Ltd., Osaka, Japan), and 1% penicillin/streptomycin (168-23191, Gibco, Grand Island, NY). We used control Colon26 cell lines together with colonies overexpressing S1P lyase (SPL) (#2, #5) and colonies with an SPL knock-out (#2, #6) for sphingolipid measurements, as previously described^27^.

In the case of cell lysates, following LC–MS/MS measurement, the areas of each sphingolipid are utilized in subsequent analysis to determine their respective levels. The protein levels of each cell lysate sample are determined using a colorimetric assay for protein concentration (DC protein assay, 500-0116, Bio-Rad Laboratories, Inc. Hercules, CA), and the areas of the corresponding internal standards are then used to calculate the final concentrations of the measured sphingolipids, reported as mg/mL.

### Sample preparation

For the measurement of all sphingolipids, serum, CSF, and urine samples were used directly without dilution. A total of 10 μL of samples were mixed with 10 μL of internal standards [SPLASH LipidoMix™, C_15_Cer1P-d7, HexCer, C_17_S1P, C_17_dhS1P, C17:1 Sphingosine (Sph), C17:1 dihydrosphingosine (dhSph), d18:1/17:0 Ceramide (Avanti Polar Lipids)] at 1 ng/mL (final concentration) and the sphingolipid contents were extracted with 100 μL of 0.1% formic acid in methanol (Wako Pure Chemical Industries) and 80 μL acetonitrile (Wako Pure Chemical Industries). The mixtures were sonicated for 5 min and then centrifuged at 16,400*g* for 10 min at 4 °C. The supernatants were then analyzed by the LC–MS/MS method.

### Sphingolipid measurement

Sphingolipids were measured using the LC–MS/MS system (LC: Acquity UPLC^®^ H-class PLUS; MS: Xevo TQ-S micro, WATERS Corporation). Briefly, 5.0 μL samples were injected and the LC separation was performed using a general-phase column [U-InertInertSustainSwift Amide 3um column: 2.1 × 100 mm (UP), GL Sciences Inc, USA ] with a gradient elution of solvent A (10 mM Ammonium acetate, 95% acetonitrile, 5% water) and solvent B (10 mM Ammonium acetate, 50% acetonitrile) at 0.6 mL/min. The conditions were as follows: A gradient run was performed at 99% solvent A and 1% solvent B for 1 min, followed by an at 90% solvent A and 10% solvent B for 1 min, next 3 min performed at 60% solvent A and 40% solvent B which followed by an at 20% solvent A and 80% solvent B for 2 min. Total run time 11 min, target column temperature 50.0a°C, target sample temperature 10.0 °C.

The mass spectrometer was operated in electrospray ionization-positive ion mode and the analytical conditions were as follows: the cone gas flow was set at 50 (L/Hr), the desolvation gas flow at 1000 (L/Hr), the source temperature at 150.0 °C, desolvation temperature at 600.0 °C.

The analyses were performed in multiple reaction monitoring (MRM) mode in the positive ion mode for sphingolipids. The MRM settings are described in Supplemental Table S1. The data were analyzed by MassLynx, TargetLynx XS (WATERS Corporation).

### Method validation

Three concentrations (0.0 ng/mL, 1.0 ng/mL, 10 ng/mL) of standard mixtures of sphingolipids [Cer (d18:1/18:0), S1P (d18:1), dhS1P (18:0), Sph (d18:1) and dhSph (d18:0)] were prepared and evaluated for the linearity of the measurements. We evaluated the intra- and inter-day precisions by conducting quintuple (5 times) measurements of the plasma, CSF, urine samples, or PBS with 1.0 and 10 ng/mL standard mixtures and calculating the coefficient of variation (CV, %). We evaluated the performance of the measurement system based on the criteria described in bioanalytical method validation guidance for the industry, published by the Food and Drug Administration in 2018, which required the CVs below 15% for the precision, except 20% at the lower limit of quantification.

### Matrix effect of serum, cerebrospinal fluid, and urine samples

The matrix effects for internal standards were examined by mixing 10 μL of internal standards at 1.0 ng/mL (final concentration) and 10 μL of PBS, plasma, CSF, or urine samples. The matrix effects for endogenous sphingolipids were examined by subtracting the peak area of intrinsic S1P, dhS1P, sphingosines, or ceramides from that of the samples to which standard S1P, dhS1P, sphingosines, or ceramides were added at 1.0 ng/mL. The peak areas of the internal standards were compared.

### Statistical analysis

Data processing and analysis were performed using R statistical software version 3.3.1 (http://www.r-project.org), and data were processed with GraphPad Prism 8.0 software (GraphPad Software, San Diego, CA). A one-way analysis of variance (ANOVA) test was used for experiments investigating the influences of the matrix. For the basic experiments in cell lysates, unpaired t-tests or multiple comparison with Tukey correction were used to analyze differences in sphingolipids. The results of the lipid levels are expressed as the means and standard deviations (SD). The results were considered significant when *p*-values were < 0.05. The *p*-values were described as * when < 0.05, ** when 0.01, *** when < 0.001.

### Supplementary Information

Supplementary Information.

## Data Availability

All relevant data are included in the manuscript, and the datasets generated or analyzed during the current study will be made available upon reasonable request. Makoto Kurano is the guarantor of this work and, as such, had full access to all the data in the study and takes responsibility for the integrity of the data and accuracy of the data analysis.
