# Supplementary material for: Development of an advanced liquid chromatography–tandem mass spectrometry measurement system for simultaneous sphingolipid analysis
Source: Sci Rep. 2024 Mar 8;14:5699. doi: 10.1038/s41598-024-56321-w (PMC10923881; doi:10.1038/s41598-024-56321-w)
Supplement: Supplementary file 1 — Supplementary Information. [file 41598_2024_56321_MOESM1_ESM.docx]

**Supplementary Table S1. MRM transition for LC-MS/MS method of sphingolipids**

| **#** | **Name** | **MRM (m/z) Parent > Daughter** | **RT** |
| --- | --- | --- | --- |
| 1 | DihydroSphingosine | 302.5 > 284.50 | 2.930 |
| 2 | DihydroSphingosine 1-Phosphate | 382.2 > 266.311 | 2.840 |
| 3 | IS DihydroSphingosine | 288.5 > 270.5 | 3.050 |
| 4 | IS DihydroSphingosine 1-Phosphate | 368.2 > 252.3 | 0.690 |
| 5 | IS Sphingosine | 286.5 > 268.4 | 2.750 |
| 6 | IS Sphingosine 1-Phosphate | 366.2 > 250.3 | 2.880 |
| 7 | Sphingosine | 300.3 > 282.31 | 2.880 |
| 8 | Sphingosine 1-Phosphate | 380.3 > 264.4 | 2.880 |
| 9 | IS_Cer 17:0 | 552.5 > 264.3 | 0.520 |
| 10 | Cer1P 12:0 | 562.42 > 264.4 | 0.550 |
| 11 | Cer1P 14:0 | 590.4 > 264.4 | 0.520 |
| 12 | Cer1P 16:0 | 618.2 > 264.42 | 0.520 |
| 13 | Cer1P 16:0 | 620.5 > 266.4 | 0.490 |
| 14 | Cer1P 18:1 | 644.5 > 264.42 | 0.520 |
| 15 | Cer1P 18:0 | 646.5 > 264.26 | 0.520 |
| 16 | Cer1P 20:0 | 674.5 > 264.42 | 0.520 |
| 17 | Cer1P 22:0 | 702.7 > 264.421 | 0.900 |
| 18 | Cer1P 24:1 | 728.6 > 264.4 | 0.900 |
| 19 | Cer1P 24:0 | 730.361 > 264.41 | 0.900 |
| 20 | Cer1P 26:1 | 756.7 > 264.42 | 0.900 |
| 21 | Cer1P 26:0 | 758.7 > 264.421 | 0.940 |
| 22 | Cer 12:0 | 482.5 > 264.3 | 0.940 |
| 23 | Cer 14:0 | 510.5 > 264.3 | 0.660 |
| 24 | Cer 14:1 | 508.5 > 264.3 | 1.200 |
| 25 | Cer 16:0 | 538.5 > 264.3 | 0.900 |
| 26 | Cer 16:1 | 536.5 > 264.3 | 1.160 |
| 27 | Cer 18:0 | 566.6 > 264.3 | 0.490 |
| 28 | Cer 18:1 | 564.5 > 264.3 | 1.200 |
| 29 | Cer 18:2 | 562.5 > 264.3 | 0.460 |
| 30 | Cer 18:3 | 560.5 > 264.3 | 0.530 |
| 31 | Cer 18:4 | 558.5 > 264.3 | 0.840 |
| 32 | Cer 20:0 | 594.6 > 264.3 | 0.460 |
| 33 | Cer 20:1 | 592.6 > 264.3 | 1.090 |
| 34 | Cer 20:2 | 590.6 > 264.3 | 0.500 |
| 35 | Cer 20:3 | 588.5 > 264.3 | 0.530 |
| 36 | Cer 20:4 | 586.5 > 264.3 | 0.810 |
| 37 | Cer 20:5 | 584.5 > 264.3 | 0.530 |
| 38 | Cer 22:0 | 622.6 > 264.3 | 0.640 |
| 39 | Cer 22:1 | 620.6 > 264.3 | 0.530 |
| 40 | Cer 22:2 | 618.6 > 264.3 | 0.530 |
| 41 | Cer 22:3 | 616.6 > 264.3 | 0.500 |
| 42 | Cer 22:4 | 614.6 > 264.3 | 0.470 |
| 43 | Cer 22:5 | 612.5 > 264.3 | 0.770 |
| 44 | Cer 22:6 | 610.5 > 264.3 | 0.880 |
| 45 | Cer 24:0 | 650.6 > 264.3 | 0.500 |
| 46 | HexCer 12:0 | 644.5 > 264.3 | 0.470 |
| 47 | HexCer 14:0 | 672.5 > 264.3 | 0.980 |
| 48 | HexCer 14:1 | 670.5 > 264.3 | 0.950 |
| 49 | HexCer 16:0 | 700.61 > 282.3 | 0.880 |
| 50 | HexCer 16:1 | 698.6 > 264.3 | 1.130 |
| 51 | HexCer 18:0 | 728.61 > 264.31 | 0.880 |
| 52 | HexCer 18:1 | 726.6 > 264.31 | 1.140 |
| 53 | HexCer 18:2 | 724.6 > 264.3 | 0.850 |
| 54 | HexCer 18:3 | 722.6 > 264.3 | 1.060 |
| 55 | HexCer 18:4 | 720.5 > 264.3 | 1.220 |
| 56 | HexCer 20:0 | 756.61 > 264.31 | 0.850 |
| 57 | HexCer 20:1 | 754.6 > 264.3 | 1.120 |
| 58 | HexCer 20:2 | 752.61 > 264.31 | 0.950 |
| 59 | HexCer 20:3 | 750.6 > 264.3 | 0.850 |
| 60 | HexCer 20:4 | 748.61 > 264.31 | 0.920 |
| 61 | HexCer 20:5 | 746.6 > 264.3 | 0.370 |
| 62 | HexCer 22:0 | 784.7 > 264.3 | 0.820 |
| 63 | HexCer 22:1 | 782.7 > 264.3 | 0.850 |
| 64 | HexCer 22:2 | 780.6 > 264.3 | 0.850 |
| 65 | HexCer 22:3 | 778.6 > 264.3 | 0.850 |
| 66 | HexCer 22:4 | 776.6 > 264.3 | 0.850 |
| 67 | HexCer 22:5 | 774.6 > 264.3 | 0.510 |
| 68 | HexCer 22:6 | 772.6 > 264.3 | 0.990 |
| 69 | SM 12:0 | 647.5 > 184.1 | 2.710 |
| 70 | SM 14:0 | 675.5 > 184.1 | 2.670 |
| 71 | SM 14:1 | 673.5 > 184.1 | 2.630 |
| 72 | SM 16:0 | 703.6 > 184.1 | 2.580 |
| 73 | SM 16:1 | 701.6 > 184.1 | 2.600 |
| 74 | SM 18:0 | 731.6 > 184.1 | 2.670 |
| 75 | SM 18:1 | 729.6 > 184.1 | 2.600 |
| 76 | SM 18:2 | 727.6 > 184.1 | 2.640 |
| 77 | SM 18:3 | 725.6 > 184.1 | 2.600 |
| 78 | SM 18:4 | 723.5 > 184.1 | 2.600 |
| 79 | SM 20:0 | 759.6 > 184.1 | 2.440 |
| 80 | SM 20:1 | 757.6 > 184.1 | 2.560 |
| 81 | SM 20:2 | 755.6 > 184.1 | 2.520 |
| 82 | SM 20:3 | 753.6 > 184.1 | 2.520 |
| 83 | SM 20:4 | 751.6 > 184.1 | 2.520 |
| 84 | SM 20:5 | 749.6 > 184.1 | 2.480 |
| 85 | SM 22:0 | 787.7 > 184.1 | 2.520 |
| 86 | SM 22:1 | 785.7 > 184.1 | 2.520 |
| 87 | SM 22:2 | 783.6 > 184.1 | 2.520 |
| 88 | SM 22:3 | 781.6 > 184.1 | 2.520 |
| 89 | SM 22:4 | 779.6 > 184.1 | 2.520 |
| 90 | SM 22:5 | 777.6 > 184.1 | 2.520 |
| 91 | SM 22:6 | 775.6 > 184.1 | 2.480 |
| 92 | 18:1 (d9) SM | 738.185 > 184.1 | 2.600 |
| 93 | LacCer 22:0 | 948.7 > 266.3 | 2.210 |
| 94 | LacCer 16:0 | 862.6 > 264.3 | 2.210 |
| 95 | LacCer 22:0 | 946.7 > 264.3 | 2.130 |
| 96 | LacCer 24:0 | 974.75 > 264.3 | 2.130 |
| 97 | LacCer 24:1 | 972.73 > 264.26 | 2.170 |
| 98 | IS_HexCer-Glu Cer C12 | 644.92 > 644.9 | 0.510 |
| 99 | IS_Ceramide1P-d7(d18:1)C15 | 531.517 > 531.51 | 0.410 |
| 100 | DHCer 14:0 | 512.4 > 494.41 | 2.680 |
| 101 | DHCer 16:0 | 540.4 > 522.41 | 2.250 |
| 102 | DHCer 18:0 | 568.4 > 550.41 | 2.740 |
| 103 | DHCer 18:1 | 566.4 > 548.41 | 2.320 |
| 104 | DHCer 20:0 | 596.4 > 578.4 | 0.600 |
| 105 | DHCer 22:0 | 624.4 > 606.41 | 0.490 |
| 106 | DHCer 22:1 | 622.4 > 604.41 | 0.600 |
| 107 | DHCer 24:0 | 652.4 > 634.41 | 0.600 |
| 108 | DHCer 24:1 | 650.4 > 632.41 | 0.600 |
| 109 | Deoxy-DHCer 14:0 | 496.7 > 268.41 | 0.710 |
| 110 | Deoxy-DHCer 16:0 | 524.7 > 268.41 | 0.600 |
| 111 | Deoxy-DHCer 18:0 | 552.7 > 268.41 | 0.490 |
| 112 | Deoxy-DHCer 18:1 | 550.7 > 268.41 | 0.490 |
| 113 | Deoxy-DHCer 20:0 | 580.7 > 268.41 | 1.170 |
| 114 | Deoxy-DHCer 22:0 | 608.7 > 268.41 | 1.170 |
| 115 | Deoxy-DHCer 22:1 | 606.7 > 268.41 | 2.020 |
| 116 | Deoxy-DHCer 24:0 | 636.7 > 268.4 | 0.440 |
| 117 | Deoxy-DHCer 24:1 | 634.7 > 268.41 | 0.440 |
| 118 | Deoxy-Cer 14:0 | 494.4 > 266.41 | 0.160 |
| 119 | Deoxy-Cer 16:0 | 522.4 > 266.4 | 0.940 |
| 120 | Deoxy-Cer 18:0 | 550.4 > 266.4 | 0.550 |
| 121 | Deoxy-Cer 18:1 | 548.4 > 266.4 | 0.550 |
| 122 | Deoxy-Cer 20:0 | 578.4 > 266.41 | 0.550 |
| 123 | Deoxy-Cer 22:0 | 606.4 > 266.41 | 0.550 |
| 124 | Deoxy-Cer 22:1 | 604.4 > 266.41 | 2.140 |
| 125 | Deoxy-Cer 24:0 | 634.4 > 266.4 | 0.440 |
| 126 | Deoxy-Cer 24:1 | 632.4 > 266.41 | 0.550 |

**Supplementary Figure S1.**

**Supplementary Figure S1. Retention times and chromatograms obtained from the MRM transitions.**

Representative samples’ (upper) and internal standards’ (lower) retention time and chromatograms of the dhSph (**A**), ceramide d18-14 (**B**), and sphingomyelin (**C**).

**Supplementary Figure S2.**

**Supplementary Figure S2. The chromatograms of sphingolipids.**

**A-L.** The chromatograms of all 12 sphingolipids measured by the new method described here are organized as follows: in each figure set, the top represents serum, the middle represents CSF, and the bottom represents urine samples.

**Supplementary Figure S3.**

**Supplementary Figure S3. Linearity analysis of sphingolipids.**

Validation of dhSph, Sph, dhS1P, S1P, and ceramides in serum (**A**), CSF (**B**) and urine (**C**) samples involved preparing standard mixtures at three concentrations (0.0 ng/mL, 1.0 ng/mL, and 10 ng/mL). Measurements were performed and evaluated for linearity. The standards included Cer (d18:1/18:0), S1P (d18:1), dhS1P (18:0), Sph (d18:1) and dhSph (d18:0).

**Supplementary Figure S4.**

**Supplementary Figure S5. Alteration of the sphingolipid levels due to SPL modulation.**

**A-G**. Each species of the Cer1P, Cer, HexCer, LacCer, dhCer, deoxy cer, deoxy dhCer and SM control Colon26 (white column), SPL overexpressing (pink column- moderately #2, cherry red column- highly expressing #5) or SPL inhibited (2 different cell lines: light blue #2 and blue columns #6) cell lines. From each type of samples, total n=5 samples were measured three times. The results are expressed as the mean ±SD.

**Supplementary Figure S4.**

**Supplementary Figure S4. Schematic graph showing alteration of the sphingolipids in SPL modulated cells.**
